# Principles of scientific research conduct, peer review, and publication: an editor’s perspective

**DOI:** 10.20517/jca.2021.37

**Published:** 2022-01-01

**Authors:** Ali J. Marian

**Affiliations:** Center for Cardiovascular Genetics, Institute of Molecular Medicine and Department of Medicine, University of Texas Health Sciences Center at Houston, Houston, TX 77030, USA.

The impetus for this perspective has been recent discussions with a few colleagues about the state of scientific research, publications, and peer review. It seems that the dictum of “publish or perish” has clouded the main objective in science, which is to discover, i.e., to gain new knowledge. It has shifted the objective to just publishing articles and/or garnering research awards, regardless of whether one makes meaningful contributions to science. The comments below are expressed with the hope of provoking discussion while emphasizing that the purpose of research is to discover, as today’s discoveries are tomorrow’s medicine.

## OPEN-MINDEDNESS

**Figure 1.**
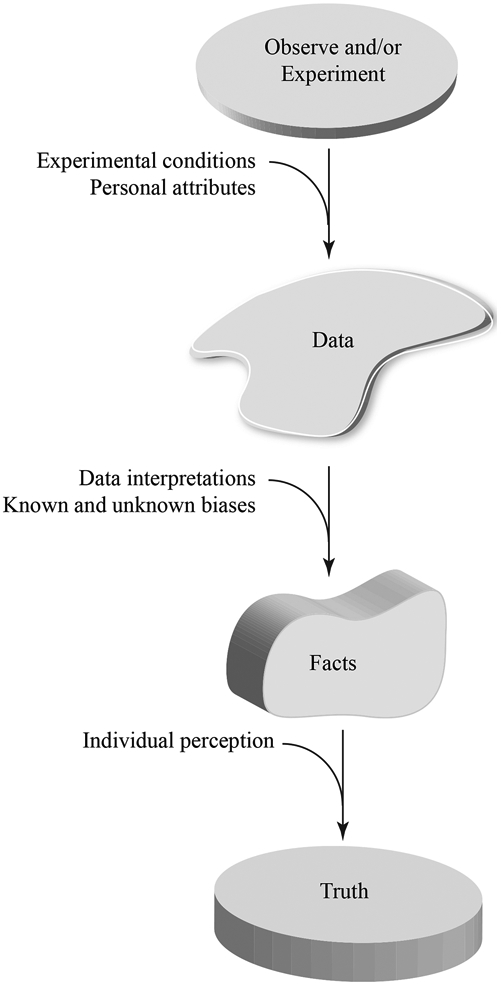
The process of reaching the truth is by gathering data through observation(s) and experimentation(s). The potential sources of errors and confounders at each step are also depicted.

Scientific discoveries are defined as findings or observations that uncover nature’s secrets. The secret in biological/biomedical sciences is how the organisms, organs, cells, or molecules work or how they are deranged in the pathological states. The ultimate goal is to discover the truth by unveiling the secrets of nature. The truth is, however, elusive to discovery, as nature shelters its secrets under layers of shields. To uncover the truth, therefore, it is essential to peel off the shields by gathering data through making observations and/or by performing experiments. The data are interpreted to extract facts, which are then used as surrogates or proxies for the truth [Figure 1]. Thus, the data are the underpinning of the scientific approach to finding the truth.

Data are often collected from observations. Solid observations have been instrumental in innumerable scientific discoveries. However, the ability to observe varies among individuals. It is partly determined by the personal attributes of the observer, which could range from possessing a prepared and educated mind to having an oblivious one. The essence of the inter-individual variability to observe is well depicted in the famous quote by Dr. Merrill Sosman: “you see only what you look for; you recognize only what you know”. Thus, the approach of gathering facts through making observations to reach the truth is indirect and imperfect.

An alternative to observation is experimentation, which is the most powerful means of gathering scientific data. The outcome(s) of each set of experiments, however, is subject to the experimental conditions. Whereas under certain conditions, a chemical or a biological reaction might work and produce valid data, it might not generate any data or generate spurious data when the experimental conditions are changed. Another important factor is the interpretation of the experimental data, which leads to the extraction of facts from the data, a process that is subject to *a priori* knowledge, expectations, and biases of the experimenter(s). Consequently, the facts, extracted from the data, are also subject to numerous confounders and cannot be considered the *sine qua non* for the truth. Therefore, all steps involved in discovering the truth - starting from making the observation(s) and gathering the experimental data to interpretation of the findings, are provisional and subject to the presence of confounders [Figure 1]. Consequently, the data, along with their interpretations, should be presented in the context of recognizing the complexity of the rules that govern nature, including biological/biomedical sciences. To quote Sir William Osler: “The processes of disease are so complex that it is excessively difficult to search out the laws which control them, and, although we have seen a complete revolution in our ideas, what has been accomplished by the new school of medicine is only an earnest of what the future has in store”.

## INTEGRITY

Integrity is essential for advancing science. The necessity is mandated by the interconnected nature of the scientific discoveries, as each scientific discovery paves the way to numerous research programs and influences the academic careers of a large number of scientists. Dishonesty in science is distinct from error. To err is human and frequent in scientific research. It is acknowledged upon its discovery and is corrected. In contrast, a departure from integrity in scientific conduct and reporting is intentional and maleficence. The departure not only could lead to defamation of the dishonest investigator but also by ushering the research in the wrong path could slow the course of the scientific advances. Such deleterious impacts also apply to scientific misconduct, such as data fabrication, duplication, intentional misinterpretation, and plagiarism. A departure from integrity in all aspects of research not only tarnishes the trust of society in science and scientists but also negatively influences the careers of the investigators, particularly those in the early stages of their academic careers. Numerous regulatory and administrative programs are implemented by the research and governmental organizations to strengthen scientific integrity. However, none alone will ever be sufficient to prevent the dishonesty of an immoral investigator, and some even could be counter-productive. Therefore, integrity, honesty, transparency, and objectivity in data acquisition, presentation, and interpretation must be constitutional to the scientists and part of the indispensable traits.

## IMPACTFUL DISCOVERIES

Scientific discoveries are generally incremental, as each discovery is made possible by the previous findings. The magnitude of the increment, however, varies. The key is to make incremental discoveries that are big enough to fulfill “the Cha-Cha-Cha theory of scientific discovery”, as described by Dr. Daniel Koshland, a distinguished former editor of Science^[1]^. Dr. Koshland described the first Cha as Charge, which pertains to discoveries by those rare people who “see what everyone else has seen and to think what no one else has thought before” (Nobel Laureate Albert Szent-Gyorgyi). The discovery of gravity by Sir Isaac Newton upon observing the movement of the planets and stars, and the discovery of low-density lipoprotein cholesterol receptors by Dr. Brown and Goldstein, upon recognizing the low-density lipoprotein cholesterol as a cause of heart attack fulfill the “Charge” discoveries. The discovery of DNA as a double-stranded helix structure by James Watson and Francis Crick was a Challenge, the second Cha of Dr. Koshland’s categories. The “Challenge” discovery is based on an accumulation of facts that are unexplainable and incongruous with the scientific theory of the time but became clear once explained. Few are fortunate enough to benefit from an impeccably prepared mind that when Chance, the third Cha, allows them to discover, they do not walk away from it. In Dr. Koshland’s depiction, the “Chance” is best exemplified by the discovery of penicillin by Sir Alexander Fleming, who, upon seeing the clear spots on a bacterial petri dish, realized that something was preventing the growth of the bacteria. The breakthroughs in science or the “Eureka” moments are novel discoveries and giant steps forward, as they markedly impact the course of science. Naturally, such discoveries are uncommon. Nevertheless, they are often the consequences of the incremental discoveries made by others. Whereas the emphasis and appreciation in scientific discoveries are on novelty, the merit of the small step discoveries, which often are considered incremental vis-à-vis the existing data, should also be recognized, as long as the data are considered robust, conclusive, and to the extent that is possible, free of biases and misinterpretations.

## SIGNIFICANCE AND TRANSLATIONAL IMPACTS

A criterion in evaluating research applications by some research funding agencies is the significance, impact, and translational aspects of the proposed studies. Likewise, many scientific journals consider the significance and translational impact in prioritizing the selection of the articles for publication. The emphasis on the impact of the discoveries and/or the translational significance of the findings seems to be excessive and should not be considered a major criterion, as long as the proposal or the discoveries are solid. It is very difficult to appreciate the significance of a finding, as the assessment is restrained by the existing knowledge. There are ample examples in the scientific literature of how the impact of discovery was not appreciated for decades. A naive mind might have asked what is the significance of knowing that DNA is a double-stranded structure, upon reading a one-page article by Watson and Crick^[2]^ published in Nature in 1953, but to Sir John Maddox, the legendary editor of Nature, the significance was self-evident. Likewise, when Friedrich Miescher isolated nuclein (DNA) from wound bandages in 1869, for 75 years, its significance was unknown, as proteins were considered the elements of inheritance and not the DNA. Through a set of simple and elegant studies, Avery *et al*.^[3]^, Hershey and Chase^[4]^, established DNA and not the protein as the hereditary material.

## HYPOTHESIS-BASED AND HYPOTHESIS-FREE RESEARCH

Scientific experiments are typically performed to test an explicit and clearly stated hypothesis, which originated based on the preliminary observations or data generated by the investigator and/or others. Naturally, the hypothesis is subject to biases, partly stemming from the existing data and partly from the investigator’s interpretation of the data and perception. To reduce bias, it is preferable to design the experiments and interpret the findings to reject the null hypothesis, i.e., the lack of an effect of an intervention or the absence of difference between (among) the experimental groups. Successful rejection of the null hypothesis then will enable one to accept the alternative hypothesis of the existence of an effect or a difference between (or among) the groups. The hypothesis-driven research has a focus, and therefore, it is best suited to address the specific question that is being tested. Hence, it has the liability of missing interesting and possibly important events that occur during experimentation but are not inclusive to the hypothesis^[5]^. The unexpected finding(s) may be noted by the investigators, but it is not typically explored because of being outside of the scope of the research hypothesis or the overall research program of the investigator. The art is to discern the experimental noise from the true unexpected finding, which could be challenging given the complexity of nature’s events. Consequently, hypothesis-driven research has the inherent shortcoming of missing potentially impactful events. The approach of focusing on the research question and not departing from the *a priori* - defined objectives is in part perpetuated by the current approach to peer review, whether in publishing the discoveries or funding the scientific project. To overcome this shortcoming, pursuing unexpected observations should be encouraged and supported.

With the advent of large-scale datasets, such as omics in biology, there has been a renewed interest in hypothesis-free research in biological sciences, which is also referred to as discovery-based research. The large data sets enable the investigators to identify patterns, interactions, correlations, and associations. The hypothesis-free approach provides the opportunity to explore and generate new hypotheses, which is inherently susceptible to the scientific mindset of the explorer as well as the *a priori* perception and biases. The point is clearly illustrated in the quote by Dr. Merrill Sosman that “you see only what you look for; you recognize only what you know”. The approach also has the potential for generating a large number of spurious findings and random associations. Consequently, the exploratory findings are provisional to being tested through hypothesis-based experiments.

## DESCRIPTIVE *VS*. MECHANISTIC STUDIES

It is a common conviction that mechanistic studies are essential to our understanding of how nature works and delineating the basis of deranged biological processes. Likewise, understanding the mechanism(s) that govern the pathogenesis of human diseases has been considered essential for developing therapies to cure the diseases. Therefore, in biological sciences, mechanistic studies are typically considered more informative and judged with a greater aura of appreciation than descriptive studies. The latter, however, is also essential and often provides the platform for mechanistic studies. Some might argue that there is no true distinction between mechanistic and descriptive studies, simply the depth of the discoveries differs between the two, with the mechanistic studies providing more in-depth explanations for the events. The Human Genome Project is an archetypal example of a descriptive study, whose findings have formed the foundation of a very large number of mechanistic studies, enabling delineating the molecular basis of a large number of human diseases.

## IMPERFECTNESS OF BIOLOGY

It merits pointing out that biological processes are noisy and seldom, if ever mathematically perfect. For example, gene transcription is pervasive, occurs across the genome, and yet the continuity is disrupted with occasional bursts of activities in response to internal or external stimuli. Likewise, transcription changes spatially and temporally within a cell, and among the same cell type in an organ, different cell types, different organs, and individuals. To quote Sir William Osler, the father of modern medicine in western society, “variability is the law of life, and as no two faces are the same, so no two bodies are alike, and no two individuals react alike and behave alike under the abnormal conditions we know as disease”. Consequently, intra-group variability in the biological data is unavoidable. However, whenever the intragroup variability is more than the anticipated effect size of the intervention or simply greater than the differences between the experimental and control groups, the biological significance of the intergroup differences becomes unclear, even though statistically might be considered significant. Such scenarios beget either changing the endpoint of the study or designing a new approach that quantifies the endpoint with less variability. Thus, it is important to recognize and appreciate the presence of often considerable variability in each set of biological data in each experimental group and not expect to generate a perfect and invariable set of biological data.

## PEER REVIEW

Given the rapid expansion of science, peer review has become a necessity in assessing the scientific research programs for funding and the research articles for publication. The process is typically fair as the reviewers are commonly committed to an impartial and fair judgment of the scientific content of the applications or papers without concerns about non-scientific issues. The reviewers are seldom grossly biased or are unsympathetic in their comments. Nevertheless, the peer-review system often places an excessive burden on the researchers to conduct experiments that might be construed as being tangential to the main purpose of the study and producing perfect data! Immunologist Ploegh^[6]^ elegantly discussed this concern in an article in Nature entitled “End the wasteful tyranny of reviewer experiments”. It is essential to recognize and end such excessive demands by the reviewers. The objective of the peer review is to increase the scientific quality, robustness, and rigor of the data while recognizing the shortcomings of each discovery.

## “LIES, DAMNED LIES, AND STATISTICS”

While it is essential to distinguish between the experimental noise and the actual outcome of the experiments, sole reliance on the statistical significance to discern the two, particularly for certain qualitative biological phenotypes, is excessive and perhaps counter-productive. It seems that the scientists have become slaves to the “*P*-value”, as the investigators stratagem all kinds of metrics to convert the biologically qualitative phenotypes to quantitative data to obtain a “significant” *P*-value. The validity of such approaches, even though widely practiced, is questionable. The two common examples in biological sciences are quantification of the immunoblots when performed on multiple membranes in independent samples, and the immunofluorescence signal on tissue sections or cells stained with an antibody against a specific protein. Notwithstanding the numerous issues that confound quantification of such qualitative phenotypes, it suffices to state that the fickleness of the “*P*-value” is evident to those who perform such quantitative comparisons. Likewise, overreliance on the “*P*-value”, when analyzing a large dataset increases the risk of spurious associations. This is a lesser concern when one uses the Bonferroni adjusted *P* values and the false discovery rates. Similarly, when analyzing large data sets with thousands or millions of data points, as in omics, it is prudent to use a sigma (standard deviation) higher than 2 and preferably a sigma of 3 or 4 to reduce the chance of a random association. Nevertheless, in analyzing the data, it is equally if not more important to consider the biological meaning of the data than the statistical significance.

## CONCLUSION

Scientists aim to discover the truth and, in the process, rely on facts that are extracted from the experimental and observational data to reach the truth [Figure 1]. The process of discovering the truth is not flawless but is unending and full of joy. This is best epitomized in the quote by Sir Winston Churchill: “Every day you may make progress. Every step may be fruitful. Yet there will stretch out before you an ever-lengthening, ever-ascending, ever-improving path. You know you will never get to the end of the journey. But this, so far from discouraging, only adds to the joy and glory of the climb”.
